# A systematic review and meta-analysis of subcutaneous onlay laparoscopic (SCOLA) approach for diastasis recti with or without mesh reinforcement

**DOI:** 10.1007/s00423-026-03985-9

**Published:** 2026-02-11

**Authors:** Antonio Vitiello, Giovanna Berardi, Roberto Peltrini, Ruggero Lionetti, Vincenzo Pilone

**Affiliations:** 1 Advanced Biomedical Sciences Department, Naples “Federico II” University, AOU “Federico II” - Via S. Pansini 5, Naples, 80131 Italy; 2 University Hospital of Naples Federico II - Via S. Pansini 5, Naples, 80131 Italy; 3 Public Health Department, Naples “Federico II” University, AOU “Federico II” - Via S. Pansini 5, Naples, 80131 Italy

**Keywords:** Diastasis recti, Ventral hernia, SCOLA, Minimally invasive surgery, Subcutaneous onlay laparoscopic approach

## Abstract

**Purpose:**

SCOLA represents a minimally invasive approach to combined diastasis recti and ventral hernia repair. This systematic review investigates the clinical efficacy and safety implications of mesh placement within this technique.

**Methods:**

We followed PRISMA 2020 guidelines to search MEDLINE and EMBASE for studies reporting SCOLA outcomes in adult patients with midline hernias and/or diastasis recti. Additionally, we retrospectively identified six institutional SCOLA cases from January 2023 to March 2025. Extracted variables included patient demographics, hernia location and size, mesh use, follow-up duration, and rates of seroma, surgical-site infection (SSI), and recurrence. We calculated pooled complication rates and performed stratified meta-analyses, estimating risk ratios (RRs) and 95% confidence intervals (CIs) for mesh vs. no-mesh cohorts.

**Results:**

Six eligible studies (1 prospective, 5 retrospective) involving 247 patients were included after systematic screening. Among all patients (99.2% laparoscopic SCOLA; mean defect size 2.77 cm; mean follow-up 9.5 months; mesh used in 72.5%), seroma occurred in 19.0% overall (21.8% vs. 11.8%; RR 1.85, 95% CI 0.88–3.89; *p* ≈ 0.104). Infections were seen only in the mesh group (5.0% vs. 0%; RR undefined, 95% CI 0.89–∞; *p* ≈ 0.058), and recurrence rates were low (1.7% vs. 0%; RR undefined, 95% CI 0.14–∞; *p* ≈ 0.318). Studies quality was moderate, limited by non-randomized designs and small observational cohorts.

**Conclusions:**

Available data suggest that SCOLA may achieve low recurrence rates, although high-quality evidence is limited. Mesh reinforcement may offer modest reduction in recurrence at the expense of an increased SSI risk. Seroma formation appears more influenced by extent of dissection than by mesh presence.

## Introduction

Diastasis recti (DR), defined as an abnormal separation of the rectus abdominis muscles along the linea alba, frequently leads to core weakness, lower back pain and postural instability (functional impairments), as well as visible abdominal bulging and dissatisfaction with body contour (cosmetic concerns) [1].

The combination of diastasis recti with midline ventral hernias presents unique challenges for conventional repair strategies and prompts consideration of the optimal way to manage both issues simultaneously. Whether through open or laparoscopic means, both plication and hernia repair approaches can be employed for DR correction; however, as minimally invasive surgery has gained traction, a variety of hybrid techniques have emerged. Champault [2] and Correa [3] were among the first to describe a videoendoscopic method that simultaneously addresses abdominoplasty, diastasis closure, and repair of small midline hernias via a limited incision and extensive subcutaneous dissection. Cuenca [4] later reported a modification using conventional laparoscopic tools to perform a comparable repair but opted to reinforce the entire area with one large polypropylene mesh sheet. Over the last decade, further variations have been introduced: Juarez Ruas (Argentina; REPA [5]: Retromuscular Endoscopic PrePeritoneal Approach), Köckerling (Germany; ELAR [6]: Endoscopic-assisted Linea Alba Reconstruction), Köhler (Austria; MILAR [7]: Minimal Invasive Linea Alba Reconstruction), Barchi [8] (Brazil; SVAWD: Subcutaneous Videosurgery for Abdominal Wall Defects), Claus & Malcher [9] (Brazil; SCOLA: Subcutaneous Onlay Laparoscopic Approach) have all published on related subcutaneous, minimally invasive techniques, reporting numerous advantages and minimal complications. Since we have performed several SCOLAs at our insistution, we aimed to review the current literature on the outcomes of this technique. To the best of our knowledge this is the first meta-analysis to focus exclusively on the SCOLA approach and to specifically evaluate whether mesh reinforcement influences both recurrence rates and postoperative infection risk.

## Materials and methods

A systematic review was conducted in accordance with PRISMA (Preferred Reporting Items for Systematic Reviews and Meta-Analyses) 2020 guidelines [10] to identify all studies evaluating subcutaneous onlay laparoscopic approach (SCOLA) repair in patients with diastasis recti associated with ventral or umbilical hernias. A completed PRISMA checklist has been submitted as supplementary material. The electronic search was carried out in MEDLINE and EMBASE using the terms “subcutaneous onlay laparoscopic repair” AND “diastasis recti.” Studies describing similar techniques but not explicitly identified by the authors as ‘SCOLA’ were excluded. Extracted data were surgical approach (laparoscopic vs. robotic), ventral defect characteristics, mesh use, complications rates, and follow-up duration.

A comparative stratified meta-analysis was performed between patients undergoing repair with versus without mesh placement. For each of the primary outcomes—seroma, infection (including surgical site and mesh infection), and recurrence—Risk Ratios (RRs) and 95% Confidence Intervals (CIs) were calculated.

The methodological quality of the included studies was assessed using the Newcastle–Ottawa Scale (NOS) [11]; This tool evaluates non-randomized studies across three domains: selection, comparability, and outcome. Each study received a total NOS score reflecting its overall risk of bias.

## Results

**Fig. 1:**
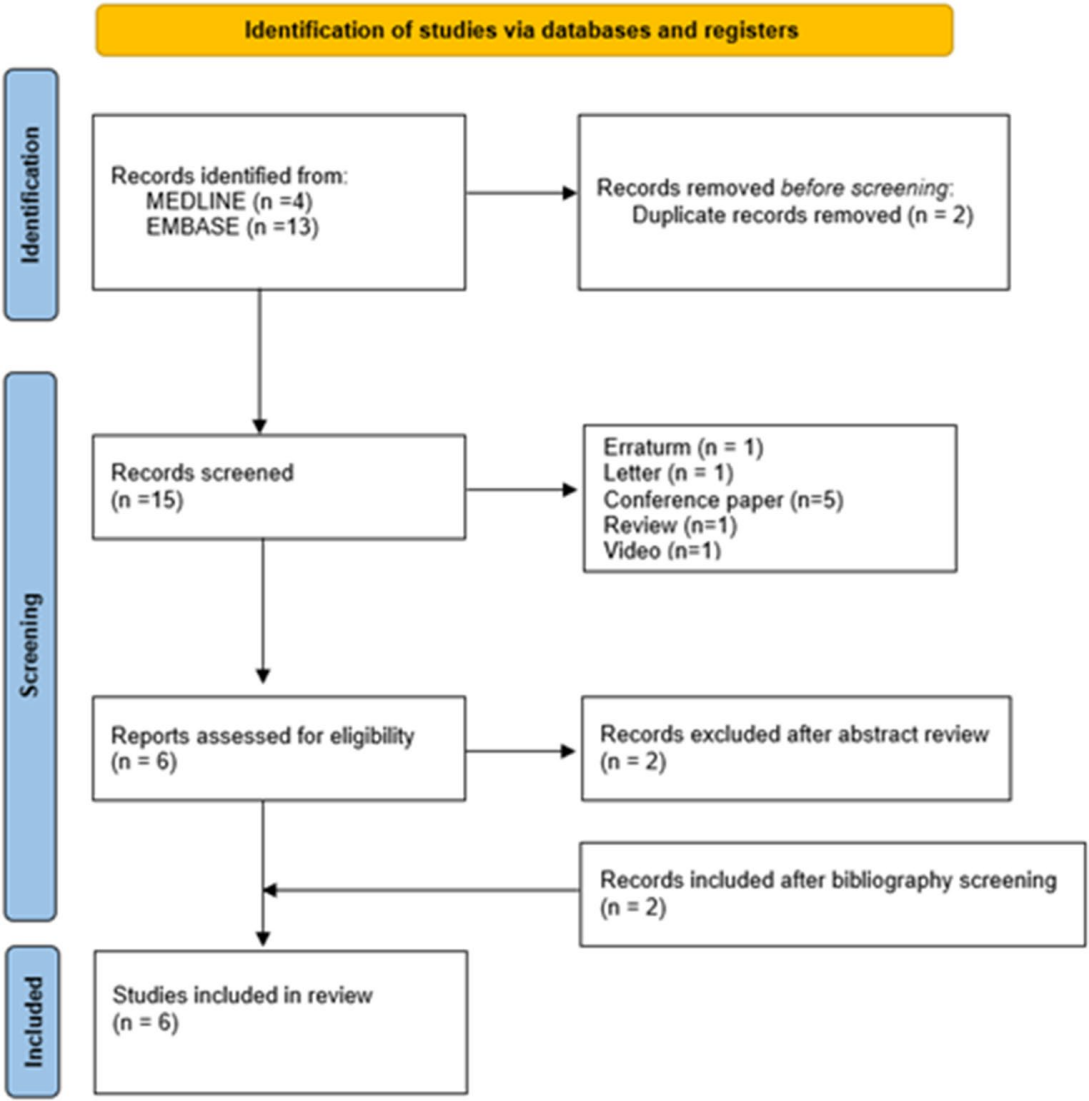
PRISMA 2020 flow diagram illustrating the identification, screening, and inclusion of studies for the review. A total of 17 records were identified, 2 duplicates removed, 15 records screened, and 6 studies ultimately included

Seventeen records were retrieved, and after removing two duplicates, 15 unique articles underwent title and abstract screening. Nine were excluded as non-original works (letters, errata, conference abstracts, videos, and reviews) and two more were discarded based on title/abstract content. Two additional studies identified through reference screening were then added. In total, six full-text articles [12–17] (1 prospective/5 retrospective; Fig. 1) met the eligibility criteria and were included in the analysis. Extracted data were reported in Table 1.

**Table 1:** Extracted data

First author & year		*N*	Surgical approach	Mesh	Mesh type	Ventral/Umbilical Hernia	Hernia types	Defect size (cm)	Complications (% and Type)	Recurrence Rate	Follow-up
Claus et al., 2018	Retrospective	48	Endoscopic pre‑aponeurotic SCOLA	Y	PP	Y	Mixed midline ventral	NR	Seroma 27% (13); SSI 2% (1); fibrotic retraction 2% (1)	2% (1)	Median 8 mo (2–19)
Dong et al., 2021	Retrospective	16	Laparoscopic 87.5% (14)/Robotic 12.5% (2)	Y	PP (7)/Self‑fixating (9)	Y	Umbilical 7; Epigastric 2; Combined 7	1.9	Seroma 18.8% (3); infected seroma 6.3% (1)	12.5% (2)	~ 2 mo (63 days)
Shinde et al., 2022	Retrospective	30	Laparoscopic SCOLA (modified)	Y	PP	Y	Umbilical & Epigastric (30)	2.10	Seroma 6.7% (2); SSI 6.7% (2); flap necrosis 0% (0)	0% (0)	3 mo
Makam et al., 2023	Retrospective	20	Laparoscopic SCOM repair	Y	PP	Y	Umbilical 10; Incisional 8; Supraumbilical 2	NR	Seroma 15% (3); electrocautery burn 10% (2); SSI 5% (1)	0% (0)	Mean 14 mo (4–25)
Mehta et al., 2024	Retrospective	33	Laparoscopic SCOLA	Y	PP	Y	Umbilical 13; Paraumbilical 16; Post‑tubal incisional 3; Epigastric 1	2.17	Seroma 45.5% (15); SSI 9.1% (3); deep mesh infection 3.0% (1); hypercapnia 6.1% (2); delayed extubation 6.1% (2)	0% (0)	4–18 mo
Kiudelis et al., 2024	Prospective	100	Endoscopic SCOLA	Y (32)/N (68)	PP	Y	Umbilical (100)	3.3	Seroma 11% (11, 8 without mesh); hypoesthesia 63% (63) (6 mo), 17% (17) (12 mo); hematoma 1% (1)	0% (0)	12 mo

*Y* YES, *N* No, *PP* PP medium weight

A total of 247 patients were included across the selected studies. Conventional laparoscopic SCOLA was the predominant surgical approach, performed in 99.2% of cases (245/247), while only 0.8% (2/247) underwent robotic-assisted repair. Purely umbilical hernias accounted for 64.8% of cases (160/247), whereas 35.2% (87/247) involved other midline or incisional defects. The weighted mean hernia defect size was 2.77 cm, and the mean follow-up across studies averaged 9.5 months (range: 2–25 months). Mesh reinforcement was used in 72.5% of repairs (179/247), whereas 27.5% (68/247) were performed without mesh.

### Overall pooled complication rates (Weighted)

Across the 247 patients included, seroma was the most frequent postoperative event, occurring in 19.0% overall. Seroma occurred in 21.8% of the mesh group versus 11.8% in the no mesh group (RR = 1.85; 95% CI: 0.88–3.89; *p* ≈ 0.104), indicating a non-significant trend toward increased risk. Infections—including superficial and deep mesh-related cases—were observed in 5.0% of patients with mesh and none in the no mesh group, resulting in an undefined RR (95% CI: 0.89–∞; *p* ≈ 0.058), suggesting a borderline association. Recurrence rates were low in both groups, with 1.7% in the mesh cohort and 0% in the no mesh cohort (RR = not estimable; 95% CI: 0.14–∞; *p* ≈ 0.318), showing no statistically significant difference. Stratified “Mesh vs No Mesh” Meta-Analysis is reported in Table 2.

**Table 2:** Stratified meta-analysis: mesh vs. no mesh

Outcome	Mesh group (*n* = 179)	No mesh group (*n* = 68)	Relative risk (RR)	95% CI	*p*-value
Seroma	39 (21.8%)	8 (11.8%)	1.85	0.88–3.89	≈ 0.104
Infections (SSI + Deep)	9 (5.0%)	0 (0%)	undefined	0.89–∞	≈ 0.058
Recurrence	3 (1.7%)	0 (0%)	undefined	0.14–∞	≈ 0.318

### Studies quality assessment

The overall methodological quality of the six included studies was moderate (Table 3). Based on the Newcastle–Ottawa Scale [18], scores ranged from 6 to 7 out of a possible 9 stars. Most studies demonstrated good participant selection and clear outcome reporting, particularly in terms of postoperative complications and follow-up duration. However, the comparability domain was generally underreported, with limited adjustment for potential confounding variables such as BMI, surgical experience, and defect size. None of the included studies were randomized, and the majority were small-scale observational cohorts. Despite these limitations, the consistency of reported outcomes and the clarity in case definitions contributed to acceptable internal validity across the dataset.

**Table 3:** Risk of bias assessment using newcastle–ottawa scale (NOS)

Study (Anno)	Selection (Max 4 ★)	Comparability (Max 2 ★)	OutcomeMax (3 ★)	Total score	Risk of bias
Claus et al., 2021	★★★★	★	★★	**7/9**	Moderate
Dong et al., 2021	★★★	★★	★★	**7/9**	Moderate
Shinde et al., 2022	★★★	★	★	**6/9**	Moderate
Mehta et al., 2024	★★★★	★	★★	**7/9**	Moderate
Makam et al., 2023	★★★	★★	★★	**7/9**	Moderate
Kiudelis et al., 2024	★★★★	★	★★	**7/9**	Moderate

## Discussion

In our recent and limited experience, minimally invasive repair of small umbilical hernias without mesh reinforcement has emerged as an intriguing option deserving closer evaluation. Between January 2024 and March 2025, we treated six patients using either a laparoscopic or robotic approach, all with small defects and without intraoperative complications. Despite the overall safety profile, we observed a non-negligible rate of postoperative seroma and one recurrence during follow-up, findings that prompted us to reflect on the real-world performance of this technique and to explore its potential role within contemporary hernia management.

Diastasis recti frequently complicates the repair of umbilical and epigastric hernias, with a prevalence of about 45% [19]. Rectus diastasis (DR) is defined as an inter-recti distance exceeding 22 mm [20], typically measured 3 cm above the umbilicus by ultrasound or calipers. Ranney’s classification grades DR width into W1 (≤ 3 cm), W2 (3–5 cm) and W3 (> 5 cm) [21]. The European Hernia Society’s midline hernia classification assigns hernia location to five anatomical zones: M1 (subxiphoidal), M2 (epigastric), M3 (umbilical), M4 (infraumbilical) and M5 (suprapubic) [22].

From a recent review of 27 studies (1,874 patients) of minimally invasive extraperitoneal repairs for ventral hernias and diastasis recti conducted between 2013 and 2023, two main laparoendoscopic strategies emerged: anterior subcutaneous onlay and posterior retromuscular/preperitoneal sublay. Anterior onlay techniques, while technically simpler and quicker (60–195 min), incur high seroma rates (up to 81%) and significant wound complications. Posterior sublay methods demand longer operative times (82–285 min) and is technically more demanding but yield low seroma incidence (0.8–7.1%) and minimal infection risk.

The Delphi consensus [23], promoted by the Italian Society of Abdominal Wall Surgery (ISHAWS) and structured around 12 recommendations, provides an operational framework for managing rectus diastasis (RAD) in post-gravidic women without excess skin. RAD is defined as an inter-recti distance > 2 cm, measurable clinically (fingerbreadth or calipers) or by ultrasound, with CT reserved for obese patients or those with associated hernias. Conservative treatment (targeted exercises) is recommended first, reserving minimally invasive linea alba plication for diastasis > 2.5 cm or after conservative failure, and adding extraperitoneal mesh reinforcement when diastasis exceeds 5 cm or a concomitant linea alba hernia > 1 cm is present. For hernias > 1 cm, a shared decision on surgery with mesh is advised. The panel also emphasized the urgent need for multicenter, long-term studies to address current evidence gaps.

In patients with rectus diastasis but minimal skin redundancy, SCOLA seems to provide effective linea alba plication via small laparoscopic ports without external scars. When moderate skin laxity coexists, hybrid methods like the Mini Abdominoplasty Minimally Invasive (MAMI) technique [24] integrate endoscopic plication, ultrasound assisted liposuction, argon plasma skin retraction, and a limited suprapubic dermolipectomy to correct both muscle separation and cutaneous excess. In cases of a frankly pendulous abdomen, a classical open abdominoplasty with wide skin excision and open rectus sheath plication remains the most appropriate intervention. Technique selection hinges on precise preoperative assessment: SCOLA for tight skin, MAMI (or SCOLA plus dermolipectomy) for mild to moderate laxity, and full abdominoplasty for pronounced ptosis. All these approaches involve extensive subcutaneous dissection and carry a high seroma risk. To mitigate fluid collections, adjunctive measures (meticulous obliteration of dead space, low pressure drains and intraoperative application of fibrin glue) [25] have proven effective in promoting tissue adherence. Patient factors such as BMI, skin quality, parity and abdominal contour must guide the reconstructive plan to balance functional repair with optimal aesthetic outcome [26].

Our pooled analysis indicated that SCOLA simultaneous repair of DR and ventral hernias achieves low recurrence rates irrespective of mesh use. Acceptable rates of seroma and infection have also been reported.

The stratified meta-analysis highlights key differences between mesh and no-mesh groups in minimally invasive ventral hernia repair. Seroma rates were higher in the mesh group compared to the no-mesh group, though this difference was not statistically significant. This finding aligns with previous studies suggesting that seroma formation is influenced by dissection extent rather than mesh presence [27].

Infection rates were exclusively observed in the mesh group, with no infections reported in the no-mesh group. This stark contrast underscores the potential risks associated with mesh use, particularly in terms of surgical site infections and deep mesh infections. The theoretical infinite RR for infections highlights the need for careful patient selection and surgical technique refinement to mitigate these risks.

Recurrence rates were comparable in the two groups, suggesting that mesh use may offer a modest advantage in recurrence prevention but requires further investigation through high-powered, prospective trials.

In our analysis, a robotic platform was employed only for two cases. While robotics offer wristed instruments, conventional laparoscopy remains preferable because the surgeon stays directly at the table, maintaining tactile feedback and spatial awareness. This direct position helps ensure a precise, symmetric plication of the linea alba during subcutaneous dissection and repair.

## Limitations

The study is limited by the predominance of retrospective designs, which may introduce selection bias. The small sample size in several studies reduces the statistical power of the analysis and infinite RR still includes the possibility of no significant difference between the groups. Short follow-up durations across studies complicates direct comparisons. Finally, the lack of standardization in surgical techniques may affect the generalizability of the findings.

## Conclusions

Available data suggest that SCOLA may achieve low recurrence rates, although high-quality evidence is limited. Mesh placement was associated with a higher risk of infection, and also seroma formation appears slightly higher in the mesh group but not statistically significant. The findings indicate that mesh use may confer a modest benefit in reducing recurrence, while potentially increasing the risk of infected seroma formation. Conventional laparoscopy remains by far the predominant approach, with robotic assistance employed in only a small minority of cases.However, the absence of statistical significance underscores the need for further investigation.

## Data Availability

No datasets were generated or analysed during the current study.
